# An Elevated Peripheral Blood Lymphocyte-to-Monocyte Ratio Predicts Favorable Response and Prognosis in Locally Advanced Breast Cancer following Neoadjuvant Chemotherapy

**DOI:** 10.1371/journal.pone.0111886

**Published:** 2014-11-05

**Authors:** Xiao-Jian Ni, Xiao-Lan Zhang, Qian-Wen Ou-Yang, Guo-Wei Qian, Lei Wang, Sheng Chen, Yi-Zhou Jiang, Wen-Jia Zuo, Jiong Wu, Xin Hu, Zhi-Ming Shao

**Affiliations:** 1 Department of Breast Surgery, Key Laboratory of Breast Cancer in Shanghai, Fudan University Shanghai Cancer Center, Fudan University, Shanghai, China; 2 Department of Oncology, Shanghai Medical College, Fudan University, Shanghai, China; 3 Institutes of Biomedical Science, Fudan University, Shanghai, China; 4 Changzhou No 2 People’s Hospital Affiliated to Nanjing Medical University, Jiangsu, China; 5 Department of Breast Surgery, the Third Hospital of Nanchang, Nanchang, Jiangxi, China; The University of Hong Kong, China

## Abstract

**Purpose:**

Neoadjuvant chemotherapy (NCT) is a standard treatment option for locally advanced breast cancer. However, the lack of an efficient method to predict treatment response and patient prognosis hampers the clinical evaluation of patient eligibility for NCT. An elevated lymphocyte-to-monocyte ratio (LMR) has been reported to be associated with a favorable prognosis for certain hematologic malignancies and for nasopharyngeal carcinoma; however, this association has not been investigated in breast cancer. The purpose of this study was to evaluate whether pre-NCT LMR analysis could predict the prognosis of patients with locally advanced breast cancer.

**Methods:**

A retrospective cohort of 542 locally advanced breast cancer patients (T3/T4 and/or N2/N3 disease) receiving NCT followed by radical surgery was recruited between May 2002 and August 2011 at the Fudan University Shanghai Cancer Center. Counts for pre-NCT peripheral absolute lymphocytes and monocytes were obtained and used to calculate the LMR.

**Results:**

Univariate and multivariate analysis revealed that higher LMR levels (≥4.25) were significantly associated with favorable DFS (*P* = 0.009 and *P* = 0.011, respectively). Additionally, univariate analysis revealed that a higher lymphocyte count (≥1.5×10^9^/L) showed borderline significance for improved DFS (*P* = 0.054), while a lower monocyte count (<0.4×10^9^/L) was associated with a significantly better DFS (*P* = 0.010).

**Conclusions:**

An elevated pre-NCT peripheral LMR level was a significantly favorable factor for locally advanced breast cancer patient prognosis. This easily obtained variable may serve as a valuable marker to predict the outcomes of locally advanced breast cancer.

## Introduction

Neoadjuvant chemotherapy (NCT) directed to the breast and axilla followed by definitive surgical therapy is considered a standard treatment option for patients with locally advanced breast cancer [1]. However, the lack of an efficient method to predict treatment response and prognosis limits the clinical evaluation of patient eligibility.

Inflammation has long been associated with cancer development, and a chronic systemic inflammatory response has been clearly implicated in the progressive process and subsequent poor outcomes of breast cancer patients [2]. Inflammation modifies the tumor biology and the quality of the immune response. Lymphocytes and monocytes are key immune cells in the inflammatory response and have been independently associated with the prognosis of various malignancies, such as breast cancer [3], gastric cancer [4], acute lymphoblastic leukemia [5], lymphoma [6], hepatocellular carcinoma [7] and nasopharyngeal carcinoma [8], [9].

Interestingly, the pretreatment lymphocyte-to-monocyte ratio (LMR) has been reported to be a prognostic factor for clinical outcomes in diffuse large B-cell lymphoma, Hodgkin’s lymphoma and nasopharyngeal carcinoma [6], [8], [10]. Therefore, we hypothesized that lymphocytes, monocytes and LMR may also play an important role in breast cancer. We performed a large-scale retrospective cohort study on locally advanced breast cancer patients and investigated the prognostic value of pre-NCT LMR for the disease. To our knowledge, this is the first large-scale study on the association of LMR and breast cancer.

## Materials and Methods

### Study population

We collected data from 542 consecutive patients who were diagnosed with locally advanced breast cancer (T3/T4 and/or N2/N3 disease) and received NCT followed by radical surgery at the Fudan University Shanghai Cancer Center (Shanghai, China) from 2002 to 2011. Data on the medical history, patient characteristics, local and distant extent of disease (evaluated by chest CT, bone scan, abdominal ultrasound, bilateral mammography, breast ultrasound or breast MRI), and the blood chemistry before NCT were collected. Core needle biopsy (CNB) was performed to confirm the diagnosis of invasive breast cancer before NCT, and fine needle aspiration of palpable or ultrasound-detected lymph nodes was performed at the time of diagnosis. Patients who had received any treatment before NCT or who had metastatic disease before surgery were not eligible for this study. Other exclusion criteria included bilateral breast cancer, male breast cancer, and inflammatory breast cancer. All patients in this study underwent an NCT regimen consisting of NE (Navelbine 25 mg/m^2^ on days 1 and 8 and epirubicin 60 mg/m^2^ on day 1 every 3 weeks), CEF (cyclophosphamide 600 mg/m^2^, day 1; epirubicin 60 mg/m^2^, day 1; and 5-fluorouracil 600 mg/m^2^, day 1; every 3 weeks) or PC (paclitaxel, 80 mg/m^2^, and carboplatin area under the curve, 2 mg min/mL, on days 1, 8, and 15 of a 28-day cycle) for a median of four cycles (range, 3–6 cycles). Mastectomy and axillary lymph node dissection were performed within 4 weeks of NCT completion, and additional cycles of chemotherapy were administered after the operation to complete a total of six cycles at the discretion of the treating physician. Radiotherapy was offered at the discretion of the treating radiation oncologist. Endocrine therapy was administered for patients with hormone receptor-positive tumors. Trastuzumab was recommended for patients with human epidermal receptor-2 (HER-2)-positive tumors in the adjuvant setting, but it was not included in any preoperative treatment. All patients were followed up every 3 months for the first year and then every 6 months until death. The last follow-up date was March 30, 2014 for all of the available patients. Our study was approved by the independent ethical committee/institutional review board of Fudan University Shanghai Cancer Center (Shanghai Cancer Center Ethical Committee). All patients provided written informed consent before inclusion in this study.

### Pathology and immunohistochemistry

All pathological evaluations were performed at Fudan University Shanghai Cancer Center. The original histological determinations were obtained through CNB before enrollment. A pathological complete response (pCR) after NCT was defined as the absence of invasive carcinoma in the breast tissue and lymph nodes of the resected specimen. Residual ductal carcinoma in situ (DCIS) was included in the pCR group. Immunohistochemistry (IHC) analysis was performed on formalin-fixed, paraffin-embedded tissue sections using standard procedures for breast tumor specimens from CNB to evaluate the expression of estrogen receptor (ER), progesterone receptor (PR) and human epidermal growth factor receptor-2 (HER-2) prior to NCT. The cut-off value for ER positivity and PR positivity was 1% positive tumor cells with nuclear staining. HER-2-positive status was defined as 3+ according to circumferential membrane-bound staining (HercepTest; Dako Cytomation) or amplification confirmed by florescent in situ hybridization. According to the 2011 St. Gallen consensus [11], we defined breast cancer subtypes as follows: luminal (ER and/or PR positive); HER-2 positive (ER and PR negative, HER-2 positive); or triple negative (TNBC; ER negative, PR negative, and HER-2 negative).

### Laboratory data

As part of the physical examinations, peripheral blood was collected before treatment, and both peripheral lymphocytes and monocytes were counted using the automated hematology analyzer Sysmex XE-5000 (Sysmex, Kobe, Japan). The peripheral LMR was calculated as the ratio of absolute counts between peripheral lymphocytes and monocytes. Baseline LMR was established from peripheral blood samples taken immediately after breast cancer diagnosis and before the initiation of any treatment modality (pre-NCT LMR). All patients had no self-reported acute infections or hematologic disorders, indicating that the cell counts could represent the normal baseline value. Finally, we excluded 12 patients with missing pre-NCT lymphocyte counts and monocyte counts, which resulted in 542 patients remaining for further analyses.

### Statistical analyses

Response was defined as complete response (CR), partial response (PR), or objective response (OR) (OR = CR+PR). Non-response was defined as stable disease (SD) or progressive disease (PD), according to WHO criteria [12] or Response Evaluation Criteria in Solid Tumors (RECIST) criteria [13]. The pCR status was assessed after surgical resection of the remaining tumor and nodes, and pCR was defined as the absence of tumor cells or absence of persistent in situ disease and negative axillary lymph nodes. All samples were assessed by two individual pathologists at Fudan University Shanghai Cancer Center.

Receiver operating characteristic (ROC) curve analysis was performed to select the most appropriate cut-off points for the lymphocyte counts, monocyte counts and LMR to stratify patients at a high risk of malignancy-related death. The score with the maximum sensitivity and specificity was selected as the best cut-off value. The endpoints assessed was disease-free survival (DFS). DFS was defined as the duration between the date of diagnosis and the date of having any of the following events: invasive recurrence in local, regional, or distant sites; a new invasive breast cancer in the contralateral breast; any second (non-breast) malignancy; or death from any cause. These endpoints were analyzed and compared using the Kaplan-Meier method and the log-rank test. Categorical variables were compared using X^2^ tests. Continuous variables, reported as lymphocyte counts, were compared using the Wilcoxon rank-sum test. Multivariate analyses with the Cox proportional hazards model were used to test independence, significance, and hazard discrimination. Covariates included in the model are given in the result tables as previously reported [14]. A two-tailed P value<0.05 was considered significant. The correlations between lymphocyte or monocyte counts and their ratio were evaluated by Spearman’s rank correlation coefficient. The above analyses were performed using the SPSS software (version 19.0, SPSS Inc., Chicago, USA).

## Results

### Patient characteristics

The clinicopathological characteristics of the cohort study are summarized in **Table 1**. All patients were female with a median age of 49 years at the time of diagnosis. After a mean follow-up time of 45.88 months, 51 of the 542 patients experienced disease recurrence. When the correlation between DFS and each clinicopathological variable was examined using univariate analysis, tumor status (HR = 1.273; 95% CI, 1.081–1.499; *P* = 0.004; **Table 2**), lymph node status (HR = 1.154; 95% CI, 1.018–1.310; *P* = 0.026; **Table 2**), grade status (HR = 0.944; 95% CI, 0.905–0.984; *P* = 0.006; **Table 2**) and NCT regimen (HR = 0.777; 95% CI, 0.648–0.932; *P* = 0.006; **Table 2**) were associated with a higher risk of recurrence and reached significance, as expected. Upon multivariate analysis, tumor status, lymph node status, pre-NCT hormone receptor status and pre-NCT HER2 status also exhibited significant association with DFS.

**Table 1:** Characteristics of 542 locally advanced breast cancer according to ALC, AMC, and the ALC/AMC ratio.

Characteristic		Overall	LMR<4.25	LMR≥4.25	*P*-value
**Age**	≤50	308	171	137	0.039^b^
	>50	234	109	125	
**Menopause status**	No	320	179	141	0.019^b^
	Yes	221	101	120	
**Tumor status**	1–2	192	97	95	0.694^b^
	3–4	350	183	167	
**Lymph node status**	0–1	400	199	201	0.135^b^
	2–3	142	81	61	
**Grade**	I	10	5	5	0.273^b^
	II	174	99	75	
	III	31	18	13	
**Stage**	I–II	202	101	101	0.551^b^
	III–IV	340	179	161	
**HR**	Negative	215	123	92	0.094^b^
	Positive	312	149	163	
**HER2**	Negative	375	199	176	0.179^b^
	Positive	147	68	79	
**Breast cancer subtype**	Luminal	190	72	118	0.000^b^
	HER2	81	45	36	
	TNBC	27	17	10	
**Response**	SD+PD	332	172	160	0.005^b^
	OR	210	108	102	
**Regimen**	NE	255	145	110	0.000^b^
	FEC	129	78	51	
	PC	158	57	101	
**Lymphocyte count (10^9^/L)**		1.57(0.1–5)*	1.48(0.1–4.8)*	1.67(0.6–5.0)*	0.002^a^
**Monocyte count (10^9^/L)**		0.40(0.1–2.1)*	0.54(0.1–2.10)*	0.26(0.1–0.7)*	0.001^a^

*Representing mean and range in the bracket; the mean LMR level was 5.01 (range, 0.13–25);

LMR, lymphocyte-to-monocyte ratio; HR, hormone status; HER2, human epithelial receptor 2.

a Wilcoxon rank-sum test;

b X2 test by two-sided Pearson’s exact test.

**Table 2:** Univariate and multivariate analyses of LMR for DFS in locally advanced breast cancer.

	Univariate analysis		Multivariate analysis	
Variable	HR (95% CI)	*P* value	HR (95% CI)	*P* value
Age	1.232(0.922–1.645)	0.158	0.998(0.623, 1.601)	0.995
Menopause status	1.282(0.959, 1.714)	0.093	1.283(0.803, 2.050)	0.297
Tumor status	1.273(1.081–1.499)	**0.004**	1.260(1.066, 1.490)	**0.007**
Lymph node status	1.154(1.018–1.310)	**0.026**	1.429(1.037, 1.969)	**0.029**
Grade	0.944(0.905–0.984)	**0.006**	0.955(0.911, 1.000)	0.051
Hormone receptor status	0.877(0.757–1.016)	0.08	0.826(0.720, 0.947)	**0.006**
HER2 status	1.056(0.992–1.124)	0.089	1.139(1.050, 1.236)	**0.002**
NCT regimen	0.777(0.648, 0.932)	**0.006**	0.927(0.752, 1.142)	0.475
LMR	0.678(0.506–0.909)	**0.009**	0.680(0.505, 0.917)	**0.011**

Bold values are significant (P<0.05). DFS, disease-free survival; LMR, lymphocyte-to-monocyte ratio; HR, hazard ratio; HER2, human epidermal growth factor receptor-2; NCT, neoadjuvant chemotherapy.

### The cutoffs of LMR, ALC and AMC pre-NCT in locally advanced breast cancer

**Figure 1:**
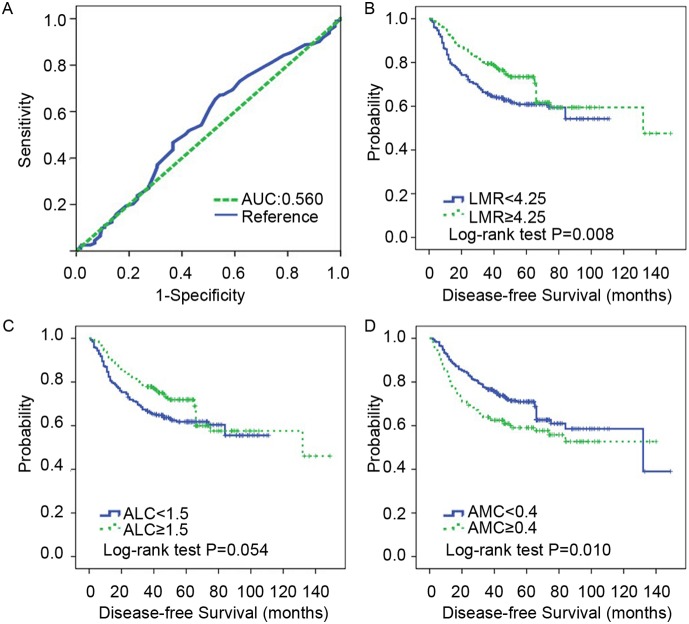
Elevated LMR indicates a favorable disease-free survival (DFS) in locally advanced breast cancer patients. A. ROC curves assessing the cut-off of LMR for predicting the occurrence of disease events in the cohort study. B. Cumulative DFS curves of locally advanced breast cancer patients with high or low LMR (number of patients, 542, number of events, 186). C. Cumulative DFS curves of locally advanced breast cancer patients with high or low ALC (number of patients, 542, number of events, 186). D. Cumulative DFS curves of locally advanced breast cancer patients with high or low AMC (number of patients, 542, number of events, 186). *LMR,* lymphocyte-to-monocyte ratio; *ALC,* absolute lymphocyte count; *AMC,* absolute monocyte count; *DFS,* disease-free survival; *ROC,* receiver operating characteristic; *AUC,* area under the curve.

The mean counts of lymphocytes and monocytes were 1.57×10^9^/L (range, 0.1–5×10^9^/L) and 0.40×10^9^/L (range, 0.1–2.1×10^9^/L), respectively. The mean LMR level was 5.01 (range, 0.13–25). ROC curve analysis was used to determine the optimal cut-off value for LMR. The LMR cut-off point for DFS was 4.25 (**Fig. 1A**), and all patients were divided into either high- (≥4.25) or low- (<4.25) LMR groups. Similarly, a lymphocyte count of 1.5×10^9^/L and monocyte count of 0.4×10^9^/L were selected as the optimal cut-off points for survival analyses. The mean lymphocyte count in the overall patients, the low-LMR group and the high-LMR group were 1.57, 1.48 and 1.67, respectively (*P* = 0.002). The mean monocyte counts in the overall patients, the low-LMR group and the high-LMR group were 0.40, 0.54 and 0.26, respectively (*P* = 0.001).

In this cohort, an association between age, menopause status, breast cancer subtype, NCT regimen, NCT response and pre-NCT LMR was observed (*P* = 0.039; *P* = 0.019; *P*<0.001; *P* = 0.005; *P*<0.001, respectively, **Table 1**). An association of high LMR level (≥4.25) and improved ORR was found (*P* = 0.005; **Table 1**).

### Elevated LMR indicates better clinical outcome in locally advanced breast cancer patients

To assess the clinical significance of pre-NCT LMR in locally advanced breast cancer, we analyzed the relationship between pre-NCT LMR and DFS. Both univariate and adjusted multivariate survival analyses revealed a significant difference between the high- and low-LMR groups. In this cohort, high-LMR cases exhibited a lower likelihood for disease events (HR = 0.678; 95% CI, 0.506–0.909; *P* = 0.009; **Table 2**) in univariate analysis and exhibited a similar trend upon multivariate analysis (HR = 0.671; 95% CI, 0.499–0.903; *P* = 0.008; **Table 2**). Additionally, patients with high LMR levels (≥4.25) generally exhibited a favorable DFS using Kaplan-Meier analysis (*P* = 0.008; **Fig. 1B**). Interestingly, high lymphocyte counts (≥1.5×10^9^/L) showed borderline significance for better DFS (*P* = 0.052), and low monocyte counts (<0.4×10^9^/L) were associated with improved DFS (*P* = 0.010; **Fig. 1C, D**). Thus, these results strongly indicate that pre-NCT LMR is directly associated with recurrent disease for patients with locally advanced breast cancer.

In addition, both lymphocyte and monocyte counts were analyzed for their independence from other covariates in the COX model (**Table S1**). LMR is not included here, considering that LMR was derived as the ratio between the lymphocyte and the monocyte counts and related to lymphocyte count (Person’s R = 0.380, *P*<0.001) or monocyte count (Person’s R = 20.766, *P*<0.001) [15]. The results showed that neither the lymphocyte nor the monocyte counts were independent factors for DFS in locally advanced breast cancer patients (**Table S1**).

## Discussion

Accumulating studies have suggested a strong link between inflammation and cancer, and pretreatment peripheral inflammatory cells, including neutrophils, lymphocytes and monocytes, have been significantly associated with prognosis in different types of cancers [9], [16], [17]. As part of the functional relevance, inflammatory responses lead to chronic oxidative stress and generate oxygen free radicals, which have been shown to stimulate cancer initiation, promotion and progression [18], [19], [20]. Moreover, tumor-associated macrophages (TAMs), an important component of inflammatory infiltrating leukocytes, may interact with tumor cells to promote tumor development by producing various cytokines and chemokines. We performed a large-scale cohort study on locally advanced breast cancer patients who received NCT to evaluate the prognostic values of peripheral lymphocytes and monocytes, together with other clinical factors. Our results confirmed previous findings that factors such as T, N, grade and overall stage were associated with a favorable prognosis for breast cancer patients. More importantly, we found that an elevated LMR was significantly associated with better ORR and DFS and, independent of other variables, was able to predict patient prognosis for locally advanced breast cancer after NCT.

Interestingly, there was a correlation between LMR and treatment regimen (*P*<0.001, **Table 1**). When the correlation between DFS and each clinicopathological variable was examined using univariate analysis, NCT regimen (HR = 0.777; 95% CI, 0.648–0.932; *P* = 0.006; **Table 2**) was associated with a higher risk of recurrence and reached significance. However, multivariate analysis did not (HR = 0.927; 95% CI, 0.752–1.142; *P* = 0.475; **Table 2**). One of the reason may be a relatively limited number of patients was used. The other could be the complex function of lymphocyte and monocyte in cancer progression. Further pre-clinical and clinical study should attempt to confirm our conclusions and clarify the underlying mechanism. For the first time, we jointly considered the counts of lymphocytes and monocytes and assessed the prognostic role of LMR for clinical outcomes in large operable or locally advanced breast cancer after NCT. However, the association of LMR with cancer survival has been reported in classical Hodgkin’s lymphoma (cHL), diffuse large B-cell lymphoma and NPC [10], [21], [22]. We found that an elevated LMR is associated with ORR in breast cancer patients receiving NCT. In addition, we found that an elevated LMR not only had a strong correlation with better DFS but was also an independent prognostic factor for survival in the multivariate analysis under the Cox model. These data support previous findings on cHL and NPC, where an elevated LMR had a significantly better OS and DFS [10], [21]. Similar results were also found in diffuse large B-cell lymphoma, which is another hematological malignancy. Together, our findings suggest that these immune cells may play similar roles in breast cancer and hematological malignancies.

Interestingly, univariate analysis revealed that a high lymphocyte count (≥1.5×10^9^/L) showed borderline significance for better DFS (*P* = 0.054), and a low monocyte count (<0.4×10^9^/L) was associated with a better DFS (*P* = 0.010; **Table S1**). These data were inconsistent with previous findings in breast cancer and other cancers, such as ovarian cancer, where a high lymphocyte count was reported as an independent favorable prognostic factor [23], [24], [25]. By contrast, pretreatment lymphopenia has been considered an indicator for poor outcomes [26], [27]. The favorable role of lymphocytes appears biologically plausible. Lymphocytes are crucial components of host immunity that are important in the destruction of residual tumor cells and related micrometastases [28], [29], and infiltrating lymphocytes can activate an effective antitumor cellular immune response [30]. Moreover, T lymphocytes could help drive cancer cells towards apoptosis due to the state of chronic activation in cancer patients [31] and lead to the death of cancer cells in response to chemotherapy by presenting tumor-associated antigens to immune cells [32], [33].

## Conclusions

Taken together, we are the first to demonstrate that pre-NCT elevated peripheral blood LMR predicts favorable response and prognosis in locally advanced breast cancer. This biomarker was directly derived from routine blood cell counts and can be easily applied in the clinical setting. We acknowledge that this finding is limited to a retrospective study in a single center, and thus, further studies performed in a multicenter or prospective manner are necessary to validate the clinical usage of pre-NCT LMR as a prognostic marker for locally advanced breast cancer.

## Ethical Standards

Informed consent was signed by each participant, and appropriate ethical committee approval was obtained. The experiments comply with the current laws of China.

## Supporting Information

Table S1 **Multivariate analysis of independent prognostic factors (n = 542).**
(DOCX)
