# Supplementary material for: An Elevated Peripheral Blood Lymphocyte-to-Monocyte Ratio Predicts Favorable Response and Prognosis in Locally Advanced Breast Cancer following Neoadjuvant Chemotherapy
Source: PLoS One. 2014 Nov 5;9(11):e111886. doi: 10.1371/journal.pone.0111886 (PMC4221197; doi:10.1371/journal.pone.0111886)
Supplement: Table S1 — Multivariate analysis of independent prognostic factors (n = 542). (DOCX) [file pone.0111886.s001.docx]

**Table S1. Multivariate analysis of independent prognostic factors (n=542).**

|  | Univariate analysis | | Multivariate analysis | |
| --- | --- | --- | --- | --- |
| Variable | HR (95% CI) | *P* value | HR (95% CI) | *P* value |
| Age | 1.232(0.922-1.645) | 0.158 | 1.028(0.636, 1.660) | 0.911 |
| Menopause status | 1.282(0.959, 1.714) | 0.093 | 1.264(0.787, 2.028) | 0.332 |
| Tumor status | 1.273(1.081-1.499) | **0.004** | 1.274(1.076, 1.507) | **0.005** |
| Lymph node status | 1.154(1.018-1.310) | **0.026** | 1.434(1.041, 1.976) | **0.027** |
| Grade | 0.944(0.905-0.984) | **0.006** | 0.955(0.911, 1.000) | 0.051 |
| Hormone receptor status | 0.877(0.757-1.016) | 0.08 | 0.837(0.729, 0.961) | **0.011** |
| HER2 status | 1.056(0.992-1.124) | 0.089 | 1.127(1.038, 1.224) | **0.004** |
| NCT regimen | 0.777(0.648, 0.932) | **0.006** | 0.937(0.759, 1.157) | 0.545 |
| Lymphocyte count | 0.752(0.563, 1.005) | 0.054 | 0.942(0.646, 1.374) | 0.756 |
| Monocyte count | 1.467 (1.094-1.967) | **0.010** | 1.353(0.917, 1.995) | 0.127 |

Bold values are significant (*P*<0.05). *DFS,* disease-free survival; *HR,* hazard ratio;

*HER2,* human epidermal growth factor receptor-2; *NCT,* neoadjuvant chemotherapy.
